# Atomic-Scale Engineering of Ge–Sb–Te Compounds: Ge Vacancies in Bulk GeSb_4_Te_7_ and Layer Sliding in GeSb_2_Te_4_ Monolayers

**DOI:** 10.3390/nano16050292

**Published:** 2026-02-26

**Authors:** Ruslan M. Meftakhutdinov, Renat T. Sibatov, Vyacheslav V. Svetukhin

**Affiliations:** 1 Scientific-Manufacturing Complex “Technological Centre”, 124498 Moscow, Russia; postbox.mrm@gmail.com (R.M.M.); v.svetukhin@tcen.ru (V.V.S.); 2 Institute of Integrated Electronics, National Research University of Electronic Technology (MIET), 124498 Moscow, Russia

**Keywords:** phase-change material, nanofilm, density functional theory, optical absorption, binding energy

## Abstract

Phase-change materials of the Ge–Sb–Te (GST) system are promising for non-volatile memory and programmable photonics owing to their reversible amorphous–crystalline transitions. Among these materials, GeSb_4_Te_7_ stands out for its optimal balance of thermal stability, switching speed, and energy efficiency. The properties of GST materials are critically dependent on structural defects, particularly germanium vacancies that occur during synthesis and operation. Using density functional theory, we demonstrate that Ge vacancies and Ge–Sb intermixing significantly influence the electronic and optical properties of GeSb_4_Te_7_. Positive binding energies reveal vacancy clustering tendencies, which systematically reduce p-type degeneracy and widen the band gap (from 0.47 to 0.67 eV at a 2.7% vacancy concentration). Consequently, the metallic optical response in the visible range diminishes, as reflected in the less negative real dielectric function. Furthermore, we extend our investigation to the fundamental building block of this material system, the GeSb_2_Te_4_ monolayer. By studying controlled interlayer displacements of Ge and Te atoms in an otherwise stoichiometric slab, we elucidate the switching mechanism in the two-dimensional limit. The pristine monolayer exhibits semiconducting behavior with an indirect band gap of 0.74 eV, while layer sliding induces a semiconductor-to-metal transition accompanied by pronounced changes in the optical absorption spectrum. The asymmetric energy barrier (1.69 eV forward, 0.60 eV reverse) suggests favorable reversible switching via structural distortions alone, without requiring chemical modifications. The obtained results, spanning from defective bulk crystals to structurally distorted monolayers, are important for the targeted optimization of GST material properties in memory devices, optical elements, and emerging nanoscale phase-change applications.

## 1. Introduction

Ge–Sb–Te (GST) phase-change materials have attracted considerable research attention owing to their exceptional property of reversibly switching between amorphous and crystalline states when subjected to thermal or electrical stimuli [1]. This rapid and non-volatile phase transition enables their application in next-generation memory technologies, particularly phase-change memory (PCM) devices [2,3], as well as in reconfigurable photonic and optical elements where programmable material states are required [4].

The properties of GST thin films are highly dependent on the deposition method [4,5,6,7]. Magnetron sputtering and pulsed laser deposition produce films with high density, typically associated with relatively few intrinsic point defects such as Ge vacancies. Step coverage (the uniformity of deposition on non-planar features such as steps, grooves, and holes) is a key metric in device fabrication. Conformal coverage, ensuring uniform thickness on vertical and horizontal surfaces, is achieved by chemical vapor deposition (CVD), atomic layer deposition (ALD), and plasma-enhanced CVD (PECVD). These methods promote surface diffusion, enhancing not only conformality but also atomic-scale ordering, including vacancy distribution and Ge–Sb intermixing. In contrast, directional deposition methods such as evaporation yield non-conformal coverage, making them preferred for lift-off processes. Importantly, deposition conditions also influence Ge vacancy clustering and interlayer sliding within the crystal structure, both of which are critical factors affecting phase-change kinetics and optical properties of GST materials.

The compound GeSb_4_Te_7_ is a promising phase-change material owing to its favorable balance of thermal stability, fast switching speed, and low energy consumption [8]. GeSb_4_Te_7_ and related GST alloys exhibit semiconductor-like band structures with interband transitions influencing their refractive index and absorption. Its functional performance is highly sensitive to structural defects, particularly germanium vacancies (V_Ge_) that intrinsically form during synthesis and device operation. These point defects behave as active centers that strongly modify key material properties, including electronic transport, optical constants, and the kinetics of the amorphous–crystalline phase transition. Defects such as Ge or Sb vacancies shift the Fermi level, potentially altering optical contrasts (in refractive index Δn and optical loss Δk) that are critical for PCM switching, although specific data on defective GeSb_4_Te_7_ are limited [9]. Intermixing of Ge and Sb atoms due to interdiffusion, which can affect phase transition characteristics, has previously been reported [10,11]. A detailed investigation of the relationship between defects and material properties is therefore essential for two primary reasons: to elucidate the fundamental structure–property linkages that govern material behavior and to provide a rational basis for the targeted optimization of GST alloys. The insights gained from such studies are critical for the informed design of next-generation high-performance PCM and programmable photonic devices [12].

Fang et al. [13] demonstrated that GeSb_4_Te_7_ thin films exhibit giant nonlinear saturable absorption under 800 nm femtosecond pulses. The effect arises from band filling near the Fermi level, enhanced by the high ${\mathrm{Sb}}_{2}{\mathrm{Te}}_{3}$ content. The nonlinear response strongly depends on film thickness, making ${\mathrm{GeSb}}_{4}{\mathrm{Te}}_{7}$ a promising candidate for ultrafast photonic devices. Wang et al. [14] showed that Se alloying in GeSb_4_Te_7_ suppresses the hole concentration via enhanced interatomic bonding (GeSe_2_ Raman modes), increasing the Seebeck coefficient and reducing thermal conductivity. This yields a peak zT=0.77 at 750 K and average zT=0.48 (300–750 K) for GeSb_4_Te_5.5_Se_1.5_, a 54% improvement over pristine GeSb_4_Te_7_.

The phase-change material GeSb_4_Te_7_ exhibits a layered structure whose fundamental building units are blocks of GeSb_2_Te_4_. Consequently, a comprehensive understanding of its functional properties requires investigation at two distinct but interconnected levels: that of the bulk crystal and that of its elementary monolayer. In the first part of this work, we focus on the bulk GeSb_4_Te_7_ system, where deviations from stoichiometry are modeled by introducing Ge vacancies. This allows us to elucidate the role of intrinsic point defects in modifying the three-dimensional electronic structure and optical response. In the second part, we isolate the behavior of the central GeSb_2_Te_4_ monolayer, the basic structural motif of the bulk compound. Here, we investigate the effect of interlayer displacements (Ge and Te layer shifts) in an otherwise stoichiometric and defect-free slab. This dual approach makes it possible to decouple the influence of chemical disorder (vacancies) from that of structural distortions (layer shifts), providing a holistic view of the mechanisms governing the electronic and optical tunability of GST-based materials from the atomic level to the nanoscale level.

Modeling of electronic transport in phase-change materials is carried out using various approaches, each of which focuses on specific aspects of the disordered structure [15,16,17,18,19]. To address the indicated interconnected levels, we perform calculations within density functional theory (DFT), an approach extensively validated for investigating defects in both bulk [20,21] and two-dimensional systems [22,23,24,25]. In the bulk GeSb_4_Te_7_ crystal, we introduce Ge vacancies at various concentrations and compute the resulting density of states, dielectric function, and optical spectra, thereby elucidating how point defects tune the three-dimensional electronic and optical properties. Concurrently, we examine the isolated GeSb_2_Te_4_ monolayer by applying controlled interlayer displacements (shifts of the Ge and Te layers) to an otherwise perfect slab.

## 2. Computational Details

Total energy calculations are performed within the DFT framework. The Projector Augmented-Wave (PAW) pseudopotentials are used to model interactions between valence electrons and ion cores. The exchange-correlation energy is treated within the generalized gradient approximation (GGA) using the Perdew–Burke–Ernzerhof (PBE) parameterization [26]. The Grimme DFT-D3 method [27] is employed to account for van der Waals interactions.

The calculations include plane waves with a kinetic energy cutoff of 520 eV. To find the optimal atomic positions, a full geometry relaxation is performed until the forces on each atom converge to a value less than 0.01 eV/Å. The convergence threshold for the total energy between successive steps of electronic minimization is set to 10^−7^ eV. Reciprocal space is discretized using k-point meshes. We use a 4 × 4 × 2 mesh for relaxation and a 6 × 6 × 2 mesh to calculate the density of states.

**Figure 1:**
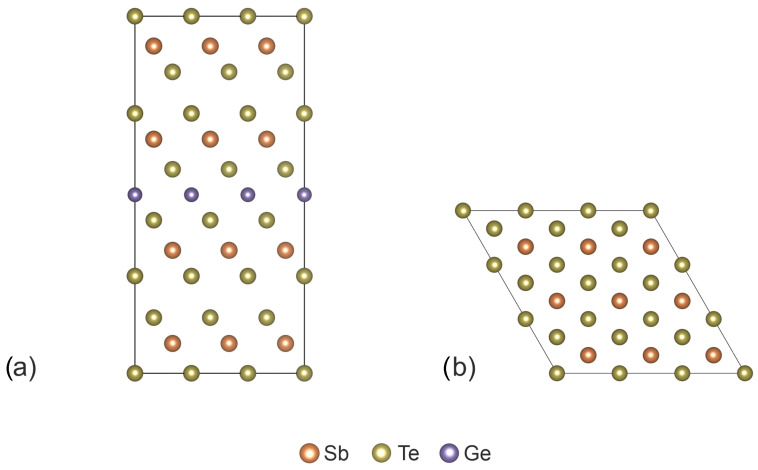
Atomic structure of the investigated GeSb_4_Te_7_ supercell: (**a**) side view and (**b**) top view.

The supercell model is used to analyze the influence of vacancies on the electronic structure and optical properties of GeSb_4_Te_7_. Figure 1 shows the studied pristine (vacancy-free) supercell after full relaxation. The supercell contains 108 atoms and has dimensions of 12.921 × 12.921 × 23.587 Å (a 3 × 3 × 1 replication of conventional hexagonal unit cells). We select the hexagonal lattice structure as the stable one. The calculated bulk lattice parameters are a=4.307 Å and c=23.587 Å, which are in good agreement with the experimental values of 4.236 Å and 23.761 Å [28], respectively. Furthermore, the Te–Te bond length calculated using the Grimme DFT-D3 correction for van der Waals interactions is 3.703 Å, which is also close to the experimental value of 3.691 Å [28]. This indicates that we have chosen a suitable method for describing GeSb_4_Te_7_.

The vacancy formation energy is calculated using the following formula:

(1) $$E_{form}=E_{V}-E_{clean}+\mu \left(A\right),$$

where $E_{V}$ and $E_{clean}$ are the total energies of the system with and without a vacancy, respectively, and μ(A) is the chemical potential of component A (A = Ge, Sb, Te), calculated as the energy per atom in the standard state of the substance.

The total binding energy between *n* objects, such as vacancies, is defined as the energy difference between two configurations: in one configuration, all objects interact, while in the other, they are sufficiently separated to be non-interacting. Thus, the total binding energy of vacancies is calculated as follows [29]:

(2) $$E_{b}\left(V_{1},V_{2},\ldots ,V_{n}\right)=\sum_{i=1}^{n}E_{V_{i}}-\left[E\left(V_{1}+V_{2}+\ldots +V_{n}\right)+\left(n-1\right)E_{clean}\right],$$

where $E_{V_{i}}$ is the total energy of a supercell with a single vacancy $V_{i}$, while $E(V_{1}+V_{2}+\ldots +V_{n})$ is the energy of a cell with the maximum possible number of vacancies.

To model the bulk GeSb_4_Te_7_ phase, characterized by three-dimensional periodicity and the presence of a complex system of point defects (vacancies and substitutional atoms), we employed the projector augmented-wave (PAW) method as implemented in the Quantum ATK package. This approach ensures high accuracy in describing the covalent–ionic bonds in the bulk crystal and reliably reproduces the formation energy of defects owing to the use of a complete plane-wave basis set for the periodic boundary problem. Given the significant computational cost of modeling large supercells with defects and the absence of a qualitative influence of spin–orbit coupling (SOC) on the main trends in the electronic properties near the Fermi level for this stoichiometry, SOC was not included in the bulk calculations.

To investigate the properties of GeSb_2_Te_4_ monolayers, we described the interactions between valence electrons and ionic cores using PseudoDojo pseudopotentials [30] within the linear combination of atomic orbitals (LCAO) basis set. The choice of this approach is justified by its computational efficiency in modeling two-dimensional systems with a large vacuum gap, as well as the possibility of a detailed analysis of chemical bonding based on localized basis functions. In the monolayer calculations, spin–orbit coupling was explicitly included. This is necessary owing to the crucial role of SOC in forming the band structure of two-dimensional topological materials based on chalcogenides, where it can lead to band inversion and the emergence of massless Dirac fermions. The exchange-correlation energy was treated within the generalized gradient approximation (GGA) using the Perdew–Burke–Ernzerhof (PBE) parameterization [26]. Van der Waals interactions were accounted for using the Grimme DFT-D3 method [27]. Structural optimization was performed until the forces acting on each atom were less than 0.001 eV/Å and the maximum energy change between successive steps was below 10^−5^ eV. To prevent spurious interactions between periodic images in the direction normal to the monolayer plane, a vacuum gap of 40 Å was employed, which significantly exceeds any possible bond length. A density grid cutoff of 120 Ha was chosen, and the Brillouin zone was sampled using a 15 × 15 × 1 *k*-point mesh. It is well established that the PBE functional tends to underestimate the semiconductor band gap; therefore, to accurately evaluate the band structure, we employed the hybrid HSE06 functional [31] including spin–orbit coupling (SOC). Note that the equilibrium geometry was obtained using the PBE-GGA functional without SOC, as SOC has a negligible influence on the structural parameters [32].

To analyze the energy barrier between the high- and low-resistance phases of GeSb_2_Te_4_, we employed the nudged elastic band (NEB) method [33] with seven intermediate images. This approach allowed us to determine the minimum energy path connecting the two configurations.

The selected combination of methods (PAW without SOC for the bulk and LCAO with SOC for the monolayer) allows an optimal balance between accurately describing the defective structure in the three-dimensional crystal and correctly accounting for relativistic effects, which are critically important for understanding the physics of the two-dimensional state.

The dielectric susceptibility tensor $\chi_{ij}\left(\omega \right)$ was calculated within the Kubo–Greenwood formalism:

(3) $$\chi_{ij}\left(\omega \right)=-\frac{e^{2}\hbar^{4}}{m^{2}\epsilon_{0}V\omega^{2}}\sum_{nm}\frac{f\left(E_{m}\right)-f\left(E_{n}\right)}{E_{nm}-\hbar \omega -i\Gamma }\pi_{nm}^{i}\pi_{mn}^{j},$$

where *V* is the volume, *f* is the Fermi–Dirac distribution function, Γ=0.05 eV is the broadening parameter, and $\pi_{nm}^{i}$ is the *i*th dipole matrix element between states *n* and *m*.

The complex dielectric function was then obtained as follows:

(4) ϵ(ω)=1+χ(ω).

The absorption coefficient α(ω) is determined by the real ($\epsilon_{1}$) and imaginary ($\epsilon_{2}$) parts of the dielectric function:

(5) $$\alpha \left(\omega \right)=\sqrt{2}\frac{\omega }{c}{\left(\sqrt{\epsilon_{1}^{2}\left(\omega \right)+\epsilon_{2}^{2}\left(\omega \right)}-\epsilon_{1}^{2}\left(\omega \right)\right)}^{1/2}.$$

This Kubo–Greenwood approach provides a rigorous framework for computing the optical response of the GeSb_2_Te_4_ monolayer, enabling us to obtain reliable absorption spectra and dielectric functions that reflect the underlying electronic structure modified by interlayer displacements.

## 3. Bulk GeSb_4_Te_7_ with Ge Vacancies

To determine which atomic vacancy is the most energetically favorable, the vacancy formation energy was calculated using Equation (1). Additionally, $E_{form}$ was computed for a supercell consisting of 48 atoms. The calculation results are listed in Table 1.

The influence of the supercell size is relatively small between supercells of 48 and 108 atoms. The largest difference is observed for the Sb vacancy and is about 140 meV. Comparing the formation energies of the three defects V_Ge_, V_Sb_, and V_Te_, we can observe that V_Ge_ has the lowest formation energy. This indicates that the GeSb_4_Te_7_ structure with V_Ge_ forms most easily and is the most stable. Therefore, all our subsequent modeling and calculations of defective systems are based on the structure containing V_Ge_.

Using Equation (2), we calculated the binding energy for two V_Ge_ defects in first- and second-nearest-neighbor configurations. For first-nearest neighbors, we obtained $E_{b}$ of approximately 0.26 eV, while for second-nearest neighbors, the binding energy is slightly larger at 0.30 eV. In both cases, $E_{b}$ is positive. According to definition (2), a negative binding energy indicates repulsion, whereas a positive binding energy signifies attraction between defects. Therefore, the GeSb_4_Te_7_ system exhibits a tendency toward V_Ge_ clustering rather than a uniform distribution. We will take this fact into account when studying systems with more vacancies.

Subsequently, the influence of different vacancy concentrations on the structure of GeSb_4_Te_7_ was investigated. We considered 0.9%, 1.8%, and 2.7% vacancy concentrations, corresponding to one, two, and three vacancies within the studied supercell. Owing to the tendency for clustering, when there were two or three vacancies in the supercell, they were positioned adjacent to each other to form a vacancy cluster.

**Figure 2:**
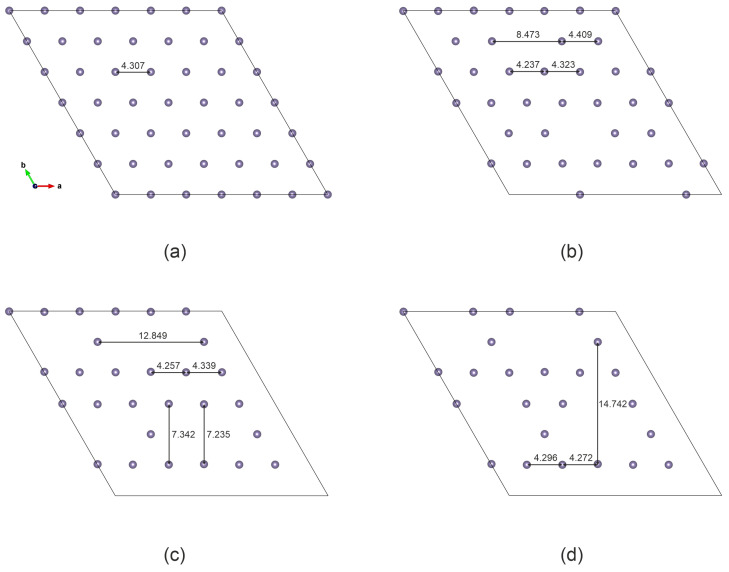
Structure of the Ge layer: (**a**) perfect structure, (**b**) structure with 1 vacancy, (**c**) structure with 2 vacancies, (**d**) structure with 3 vacancies. The indicated distances between atoms are given in angstroms.

First, we examine the structure of GeSb_4_Te_7_ with Ge vacancies. The distance between the germanium layer and the adjacent tellurium layer decreases slightly from 1.682 Å for the perfect structure to 1.656 Å for the structure with three vacancies. The Ge–Te bond length changes. If in the ideal lattice it is 3.002 Å, then for the structure with three vacancies in different parts of the pore it varies from 2.976 Åto 2.896 Å. As expected, the Ge layer itself undergoes the strongest rearrangements (see Figure 2). In the pure structure, the distance between Ge atoms is 4.307 Å. When one Ge is removed, a small hexagonal pore is formed, with dimensions of 8.473 Å (see Figure 2b), which is less than two Ge–Ge distances in a perfect supercell. This indicates a tendency to decrease its size. In addition, near the vacancy, the Ge–Ge distance decreases to 4.237 Å. A divacancy forms an elongated pore with a length of 12.849 Å, its width in the middle is smaller than at the edge (Figure 2c). A trivacancy creates a triangular pore. In pristine GeSb_4_Te_7_, the distance between the Ge layer and the adjacent Te layer is 1.68 Å. Introducing Ge vacancies progressively reduces this distance—to 1.67 Åfor one vacancy, 1.65 Åfor two vacancies, and 1.64 Åfor three vacancies.

**Figure 3:**
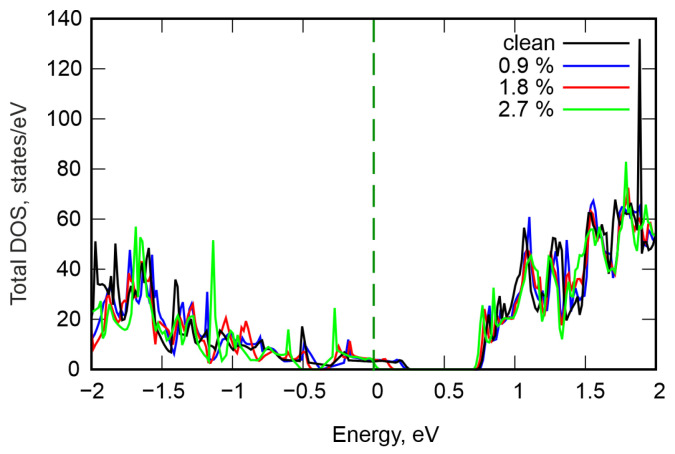
Total density of states of GeSb_4_Te_7_ for different Ge vacancy content. The Fermi level is set at 0 eV.

**Figure 4:**
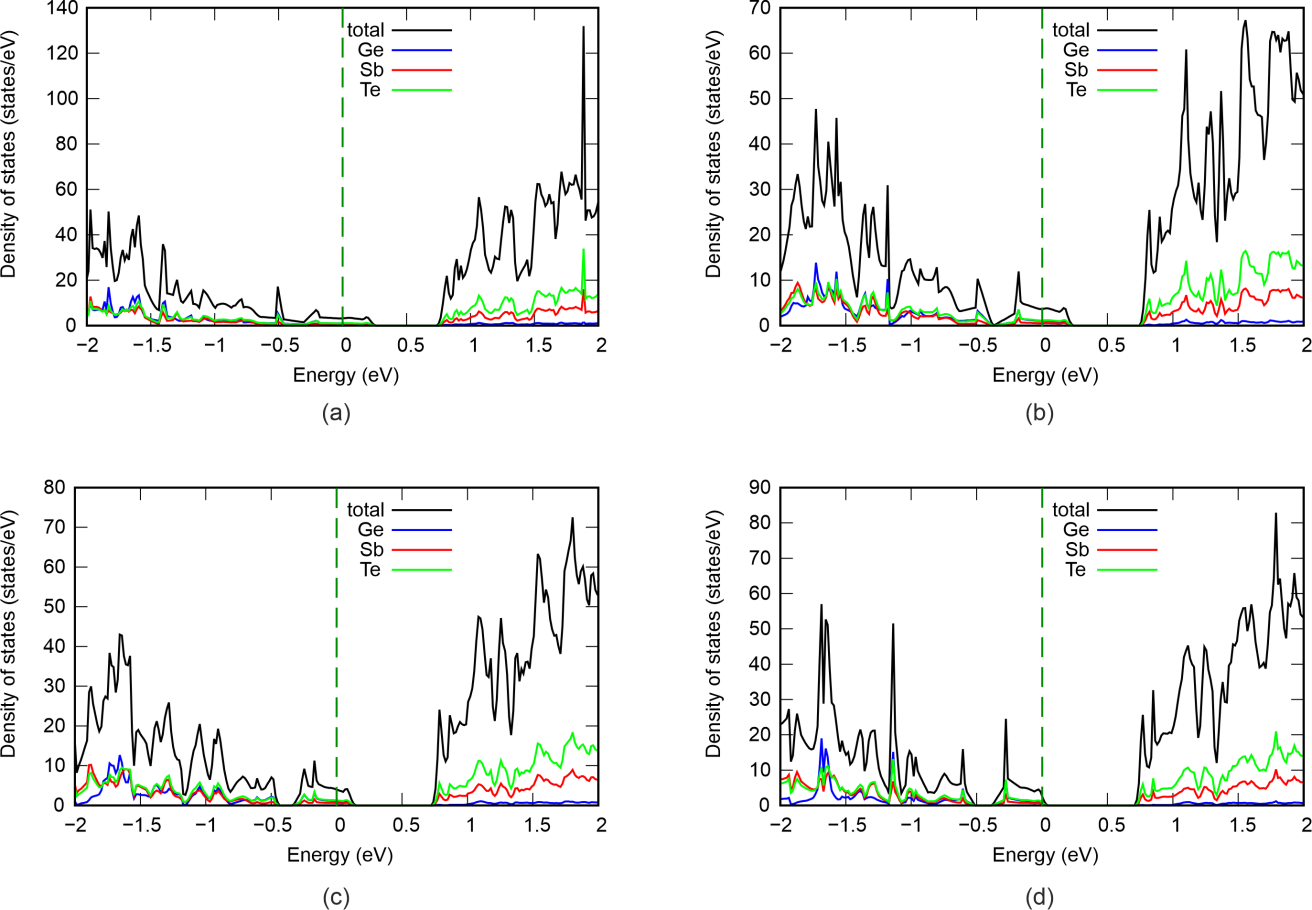
Projected density of states of GeSb_4_Te_7_: (**a**) defect-free structure, (**b**) 0.9% Ge vacancy content, (**c**) 1.8%, (**d**) 2.7%. The Fermi level is set at 0 eV.

Figure 3 shows the total density of electronic states in structures with different numbers of vacancies. It can be seen that the pristine structure is a strongly degenerate p-type semiconductor with a band gap of 0.47 eV. Our results are in close agreement with previous studies [8,14]. Increasing the vacancy concentration causes the band gap to increase to 0.67 eV at Ge vacancy content of 2.7%. Concurrently, the valence band maximum (VBM) shifts toward lower energies and moves closer to the Fermi level, resulting in reduced degeneracy. The conduction band minimum (CBM) remains virtually unchanged. Furthermore, we calculated the projected density of states, shown in Figure 4. It can be observed that the VBM and CBM are formed by the electronic states of all atoms; however, the largest contribution comes from Te. The gap change is attributed primarily to lattice distortions in adjacent Ge and Te layers and changes in the Ge–Te bond length. The resulting stresses reduce the electron energy and VBM.

**Figure 5:**
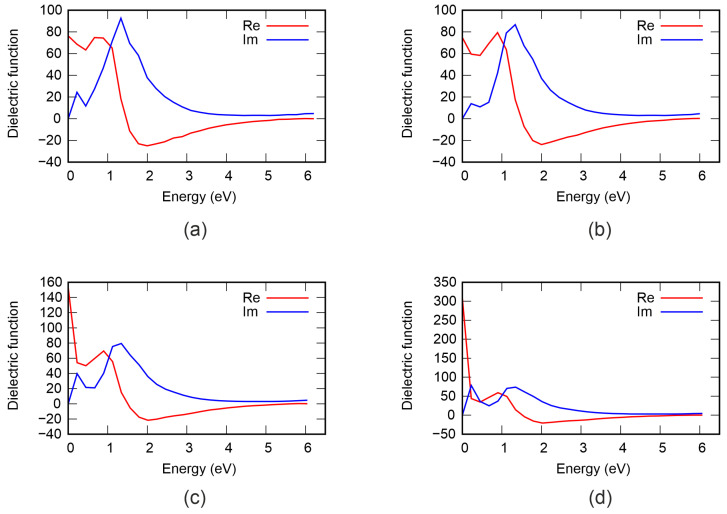
Dielectric function of GeSb_4_Te_7_: (**a**) defect-free structure, (**b**) 0.9% Ge vacancy content, (**c**) 1.8%, (**d**) 2.7%.

Within the independent-particle approximation and neglecting local field effects, we calculated the dielectric function ε for pristine GeSb_4_Te_7_ and for structures with different vacancy concentrations. The real and imaginary parts of ε are shown in Figure 5. A drop in Re(ε) is observed in the low-energy part of the visible range, indicating negative dispersion, which is characteristic of materials with a tendency toward metallic behavior. Furthermore, at 1.55 eV (for defect-free GeSb_4_Te_7_), Re(ε) changes sign and becomes negative. As is well known, negative values of the real part of the dielectric function are characteristic of materials with metallic properties, where the screening of external fields is incomplete and free-carrier response dominates. The minimum of Re(ε) is reached at approximately 2 eV. Notably, the minimum value of Re(ε) becomes less negative with increasing vacancy concentration, which is consistent with our previous conclusion that GeSb_4_Te_7_ becomes less degenerate (i.e., exhibits lower free-carrier concentration) as the number of vacancies increases. This trend reflects the gradual suppression of metallic character upon introduction of structural defects. Additionally, we note that a high oscillator strength and weak damping contribute to condition Re(ε)<0, which is essential for potential plasmonic applications in the visible range.

The imaginary part of the dielectric function characterizes the material’s ability to absorb energy from an electromagnetic field and is directly related to dielectric losses, which represent the dissipation of energy within the material as heat. For defect-free GeSb_4_Te_7_, strong absorption is not observed up to 0.5 eV owing to weak interband transitions. The only small peak in Im(ε) in the low-energy region is associated with intraband (free-carrier) absorption, which is typical for degenerate semiconductors. When the photon energy exceeds approximately 0.5 eV, absorption increases significantly owing to the onset of direct transitions from the valence band to the conduction band. After reaching a maximum, Im(ε) monotonically decreases with increasing photon energy, following the general trend expected for interband absorption in semiconductors. It is worth noting that the onset of the absorption decrease correlates closely with the point where Re(ε) changes sign, suggesting a link between the optical transition thresholds and the dielectric response regime. For GeSb_4_Te_7_ with different vacancy concentrations, our conclusions remain qualitatively unchanged, although the magnitude of absorption and the exact positions of features shift slightly owing to modifications in the electronic band structure.

**Figure 6:**
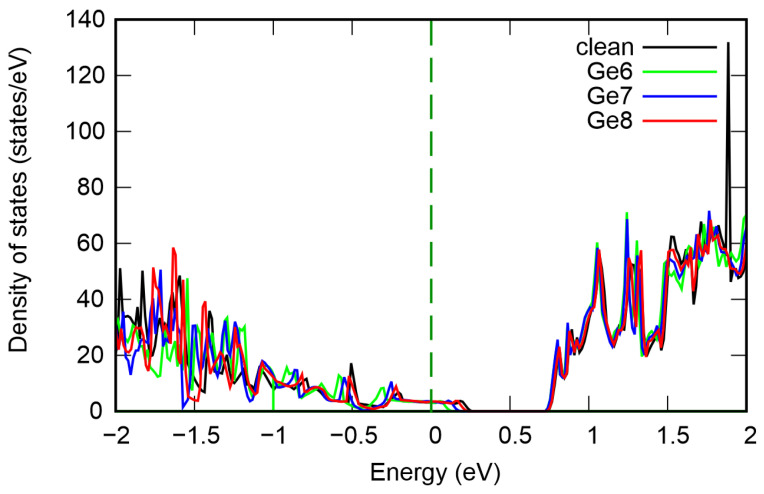
Total density of states of GeSb_4_Te_7_ with varying degrees of Ge and Sb mixing in the Ge–Sb sublattice. The Fermi level is set to 0 eV.

**Figure 7:**
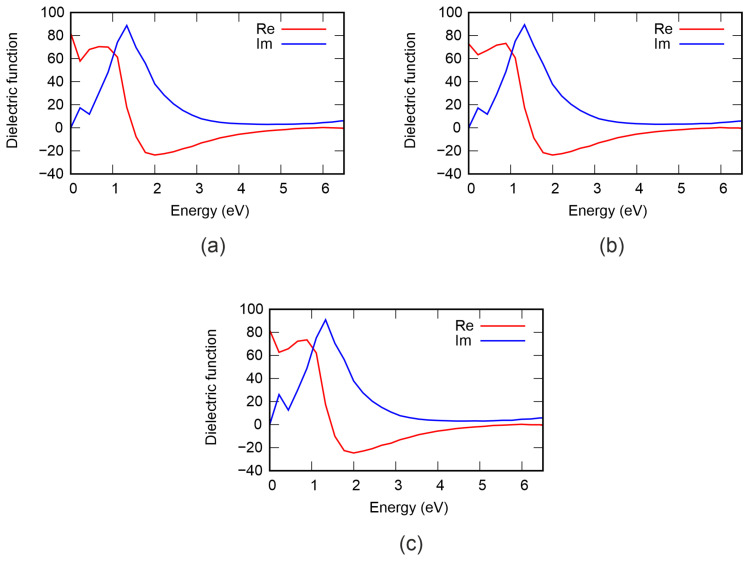
Dielectric function of GeSb_4_Te_7_ with varying degrees of Ge and Sb mixing in the Ge–Sb sublattice: (**a**) Ge6, (**b**) Ge7, (**c**) Ge8.

In the next step, we study the effect of diffusion mixing of atoms in the Ge–Sb sublattice on the electronic and optical properties. In an ideal supercell, the Ge layer contains 9 atoms. The structures labeled Ge8, Ge7, and Ge6 indicate the exchange of one, two, and three atoms between the Ge and Sb layers, respectively. The numeral in each label denotes the number of Ge atoms remaining in the Ge layer. Figure 6 shows the total density of states of GeSb_4_Te_7_ with varying degrees of Ge and Sb mixing. It can be seen that intermixing of Ge and Sb atoms causes the valence band maximum (VBM) to shift to lower energies, thereby increasing the fundamental band gap to approximately 0.58 eV. This band-gap widening is attributed to the modification of local bonding environments and the reduction of structural-disorder-induced gap states. The dielectric function of GeSb_4_Te_7_ for different degrees of Ge and Sb mixing in the Ge–Sb sublattice is shown in Figure 7. The Re(ε) and Im(ε) curves are qualitatively similar to those obtained for the perfect structure and for vacancy-containing models. However, there is one interesting feature: alternating increases and decreases in the static dielectric constant Re(ε(0)) are observed. For the ideal lattice, Re(ε(0)) is 76.31; it then exhibits an alternating pattern: 81.87 for Ge8, 72.88 for Ge7, and 81.91 for Ge6. In addition, the imaginary part of the dielectric function remains essentially unchanged. This behavior is expected, as Ge–Sb mixing preserves both the composition and the crystal structure of GeSb_4_Te_7_, leaving the absorption characteristics unaltered.

## 4. Switching Behavior of the GeSb_2_Te_4_ Monolayer

**Figure 8:**
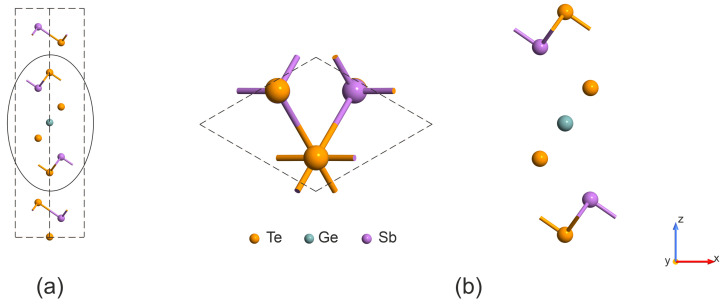
The crystal structures of (**a**) bulk GeSb_4_Te_7_ and (**b**) a GeSb_2_Te_4_ monolayer.

The layered structure of GeSb_2_Te_4_ crystallizes in a hexagonal unit cell formed by a seven-atom slab, as shown in Figure 8a. The slabs are held together by van der Waals forces. The exfoliation energy is approximately 25 meV/Å [32], indicating that they can be readily separated. The crystal structures of bulk GeSb_4_Te_7_ and a GeSb_2_Te_4_ monolayer are shown in Figure 8.

**Figure 9:**
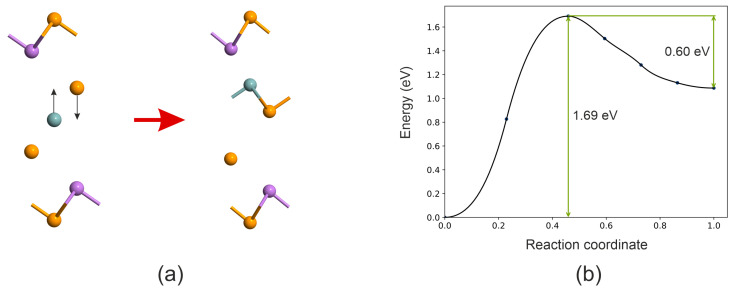
(**a**) Switching path between Configuration 1 (initial state) and Configuration 2. Te atoms are colored orange, Ge atoms grey-blue, and Sb atoms purple. (**b**) Switching barrier calculated from the NEB method.

Figure 8b displays the geometric structure of the GeSb_2_Te_4_ monolayer, which can be obtained by exfoliating a block from bulk GeSb_4_Te_7_. Our work is inspired by Ref. [34], which models the switching mechanism involving a phase transition between low- and high-resistance states in GeSbTe chalcogenide superlattices. It was shown that the primary transition mechanism involves a vertical displacement of the Ge layer through the Te layer, followed by lateral movement of the GeTe sublayer toward the low-energy final structure. Such sliding is possible because of the weak van der Waals forces holding the layers together. In the GeSb_2_Te_4_ monolayer, van der Waals forces are absent [32]; therefore, we consider only the displacement of Te and Ge layers, as illustrated in Figure 9a. The switching barrier from Configuration 1 (initial state) to Configuration 2, calculated using the NEB method, is 1.69 eV (Figure 9b). For the reverse transition back to the initial state, the barrier height is significantly lower, at 0.60 eV. These results confirm that the switching mechanism in the isolated monolayer involves a distinct asymmetry in the energy landscape, favoring reversible transitions between configurations via layer displacements.

**Figure 10:**
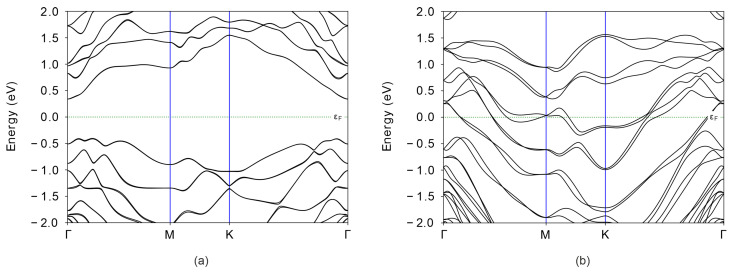
Band structure of GeSb_2_Te_4_ (**a**) before and (**b**) after displacement of Ge and Te layers.

**Figure 11:**
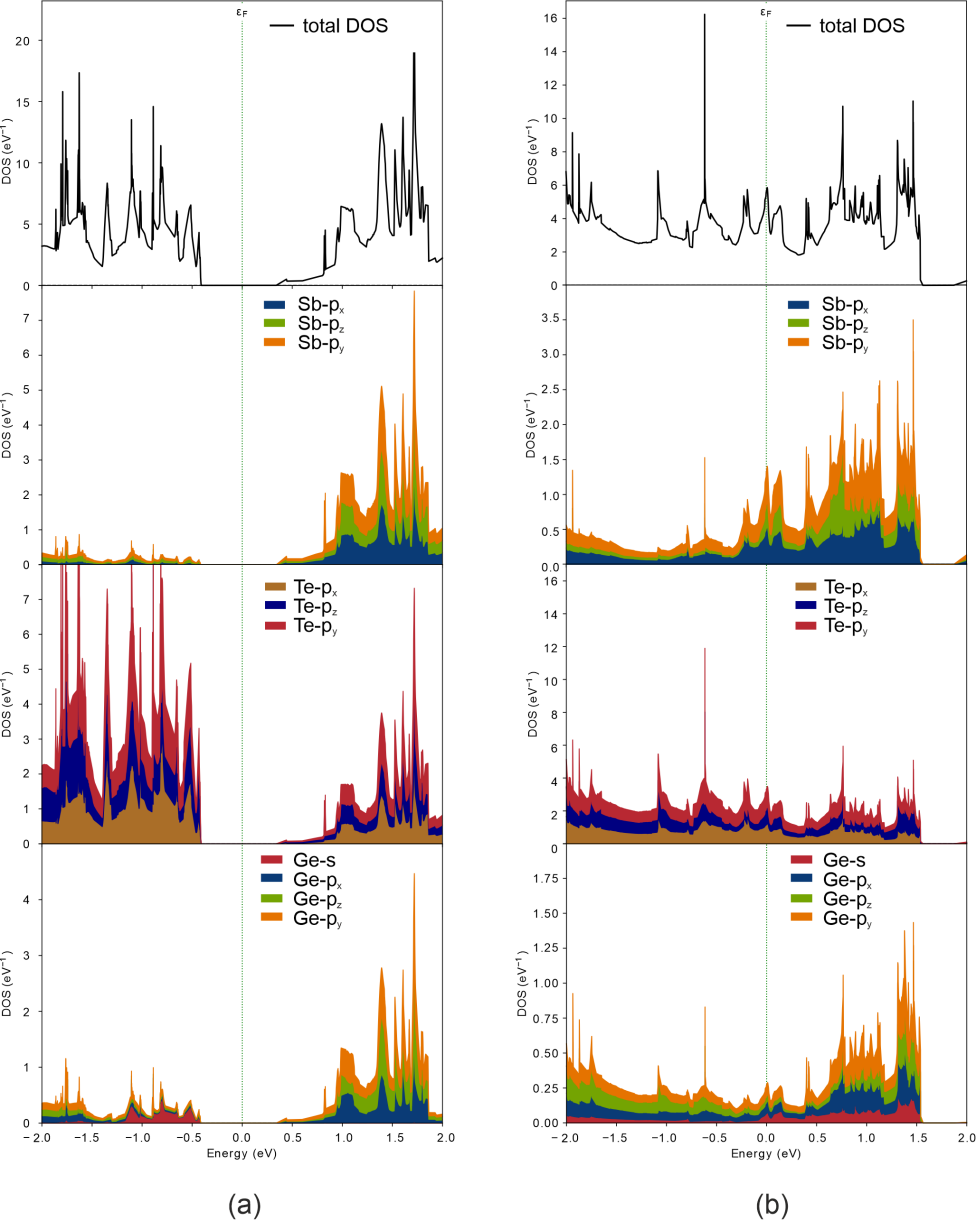
Projected density of states (PDOS) of GeSb_2_Te_4_ (**a**) before and (**b**) after displacement of Ge and Te layers.

To validate our assumption that different configurations of GeSb_2_Te_4_ exhibit distinct conductivity, we investigated its electronic properties by computing the band structure and density of states. Figure 10 presents the band structure and density of states for both pristine and modified GeSb_2_Te_4_. Prior to Ge and Te displacement, GeSb_2_Te_4_ is an indirect-band-gap semiconductor with a band gap of 0.74 eV (Figure 10a). The valence band maximum (VBM) is located in the valley between the M and Γ high-symmetry points. Furthermore, analysis of the density of states reveals that the VBM is primarily dominated by Te *p*-electrons (Figure 11a). The conduction band minimum (CBM) is situated at the Γ point (Figure 10a) and arises mainly from Sb and Te *p*-states. Additionally, the *p*-states of Ge and Te exhibit strong hybridization and remain degenerate over most of their extent (Figure 11a), indicating the covalent character of the bonds between Ge and Te atoms. In contrast, the chemical bonding between Sb and Te displays both covalent and ionic character. Covalent bonding is evidenced by the strong hybridization of Sb and Te *p*-states, while ionic bonding is suggested by the asymmetric distribution of states below and above the Fermi level: Te states predominantly occupy energies below $\varepsilon_{F}$, whereas Sb states dominate above $\varepsilon_{F}$. Similar conclusions were drawn from electron localization function analysis in Ref. [32].

The displacement of Ge and Te layers induces a phase transition in GeSb_2_Te_4_. Following this modification, the material exhibits metallic-like conductive properties (Figure 10b). This dramatic change in electronic structure confirms that layer sliding effectively tunes the conductivity, offering a mechanism for resistance switching in the monolayer regime.

**Figure 12:**
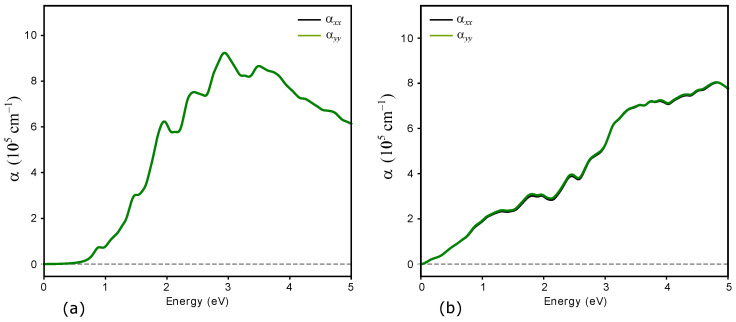
Absorption spectra of GeSb_2_Te_4_ (**a**) before and (**b**) after displacement of Ge and Te layers. In the pristine configuration (**a**), the absorption onset occurs at approximately 0.7 eV, which coincides with the fundamental band gap and corresponds to transitions from the valence band to the conduction band. The absorption edge is characteristic of semiconducting behavior. After layer sliding (**b**), the spectrum undergoes a dramatic transformation: the absorption edge disappears and significant absorption extends to low photon energies, confirming the semiconductor-to-metal transition induced by structural distortion.

Figure 12 presents the optical absorption spectra of GeSb_2_Te_4_ before and after displacement of the Ge and Te layers. Both phases exhibit strong absorption. In Configuration 1, GeSb_2_Te_4_ begins to absorb at an electromagnetic radiation energy of approximately 0.7 eV, which coincides with the band-gap value and indicates transitions from the valence band to the conduction band. After layer sliding (Configuration 2), the absorption edge disappears and the spectrum exhibits metallic-like behavior, with significant absorption extending to low energies, consistent with the semiconductor-to-metal transition observed in the electronic structure. These optical characteristics highlight the potential of layer displacement for modulating the optoelectronic response of GST-based monolayers in nanoscale devices.

Extending the multiscale modeling framework introduced by Pela et al. [35] represents a promising future direction, offering the potential to predict how atomic-scale defects govern functional properties in phase-change materials and to directly connect theoretical calculations with experimental device performance.

## 5. Concluding Remarks

The functional properties of GeSb_4_Te_7_ materials are critically dependent on structural defects. DFT-based calculations demonstrate that germanium vacancies (V_Ge_) are the most stable intrinsic point defects in GeSb_4_Te_7_. The calculated positive binding energy between vacancy pairs confirms their tendency to form clusters rather than remain uniformly distributed. The incorporation of V_Ge_ clusters systematically modifies the electronic structure, reducing p-type degeneracy and increasing the band gap from 0.47 eV to 0.67 eV at a 2.7% vacancy concentration. These electronic changes correspondingly affect the optical properties, particularly diminishing the metallic character in the visible range as evidenced by the decreasing negative values of the dielectric function’s real part. These results establish V_Ge_ concentration as a crucial parameter for controlling the functional properties of GeSb_4_Te_7_ in phase-change memory and photonic applications.

The investigation of the GeSb_2_Te_4_ monolayer demonstrates that interlayer displacements of Ge and Te atoms provide an effective mechanism for tuning its electronic and optical properties. Switching between configurations involves an asymmetric energy landscape, with a forward barrier of 1.69 eV and a significantly lower reverse barrier of 0.60 eV, suggesting favorable reversible transitions under external stimuli. The pristine monolayer is an indirect band gap semiconductor (0.74 eV), with the valence band maximum dominated by Te *p*-states and the conduction band minimum arising from hybridized Sb and Te *p*-states. Analysis of the projected density of states reveals strong Ge–Te *p*-state hybridization indicative of covalent bonding, while Sb–Te bonding exhibits mixed covalent and ionic character.

Upon layer displacement, the material undergoes a semiconductor-to-metal transition, with the modified configuration displaying metallic-like conductivity and finite states at the Fermi level. This electronic transformation is accompanied by a dramatic change in optical response: the pristine monolayer exhibits an absorption onset at approximately 0.7 eV corresponding to band-to-band transitions, whereas the displaced configuration shows metallic-like absorption extending to low photon energies. The strong contrast in both electronic and optical properties between configurations highlights the potential of layer sliding as a switching mechanism for nanoscale GeSb_2_Te_4_ PCM.

## Figures and Tables

**Table 1:** Vacancy formation energy $E_{form}$ (eV).

	V_Ge_	V_Sb_	V_Te_
48 atoms	0.85	1.80	1.81
108 atoms	0.82	1.66	1.81

## Data Availability

The original contributions presented in this study are included in the article. Further inquiries can be directed to the corresponding author.

## Abbreviations

The following abbreviations are used in this manuscript:

DFT	density functional theory
PCM	phase-change memory
GST	Ge–Sb–Te
DOS	density of states
PBE	Perdew–Burke–Ernzerhof parameterization
HSE06	Heyd–Scuseria–Ernzerhof (2006) functional
GGA	generalized gradient approximation
CVD	chemical vapor deposition
ALD	atomic layer deposition
PECVD	plasma-enhanced chemical vapor deposition
LCAO	linear combination of atomic orbitals
SOC	spin–orbit coupling
PAW	projector augmented-wave method
